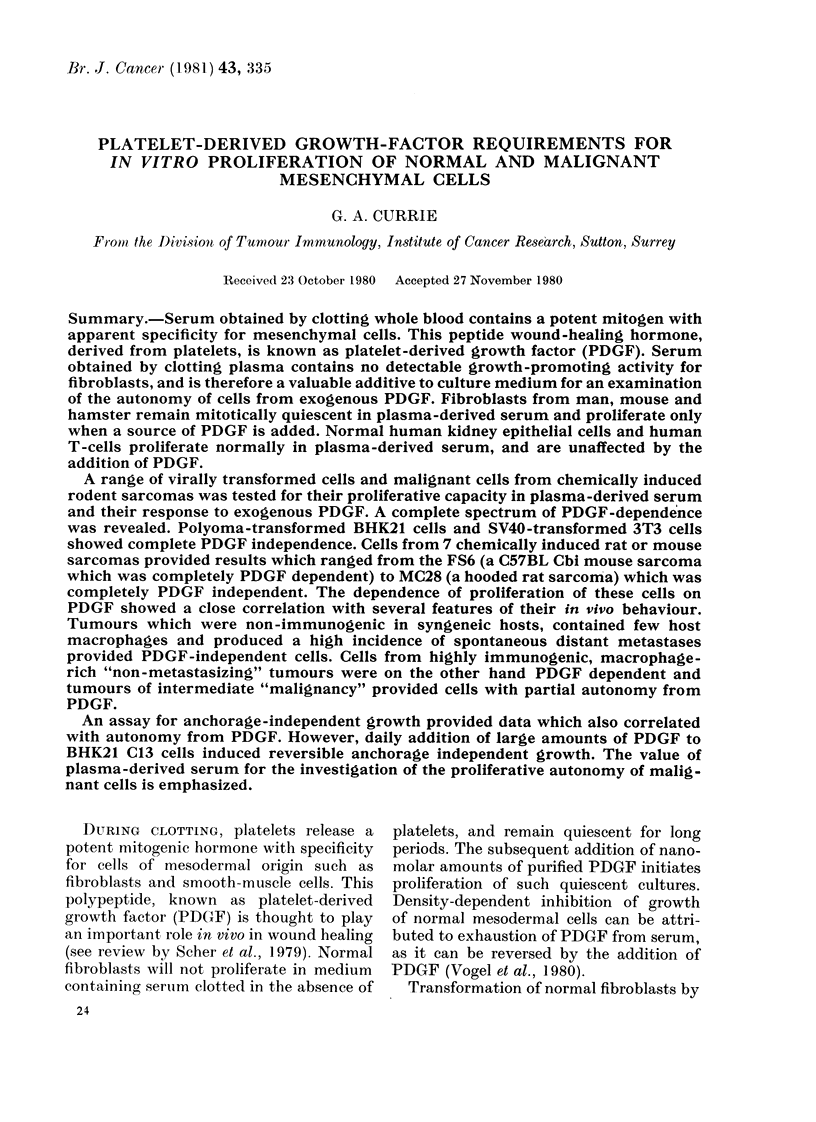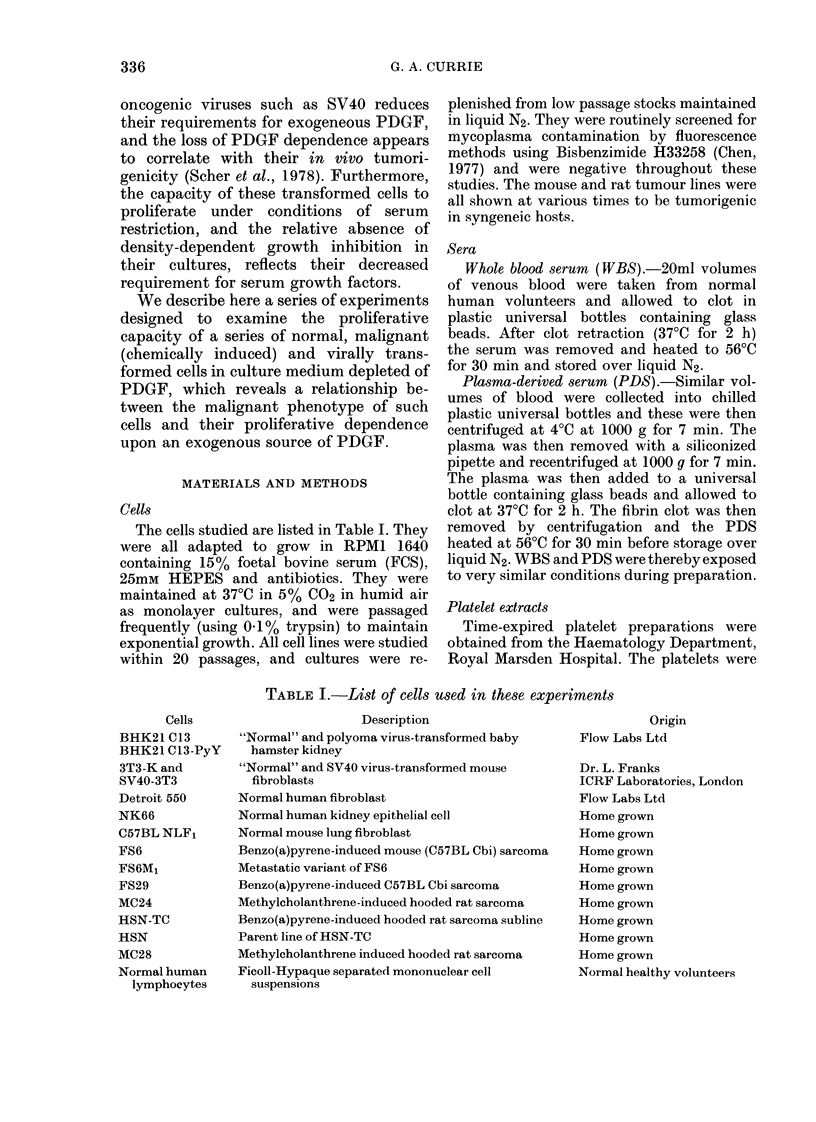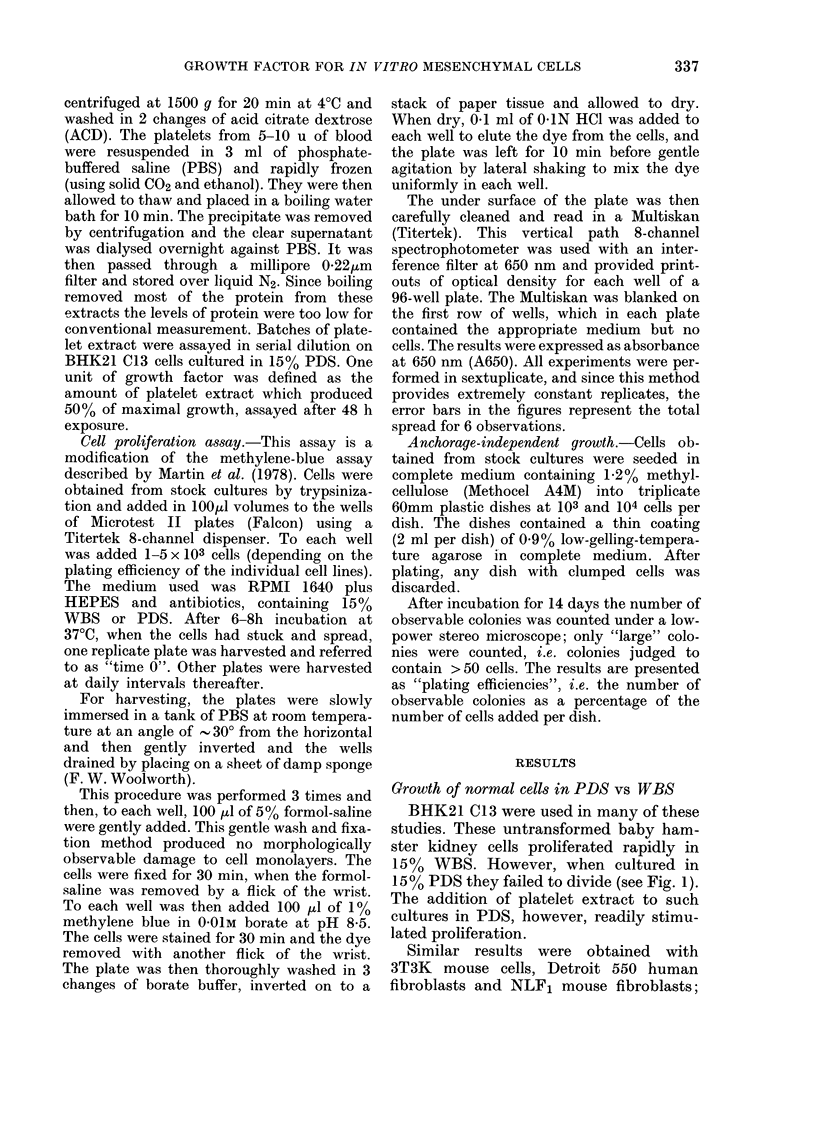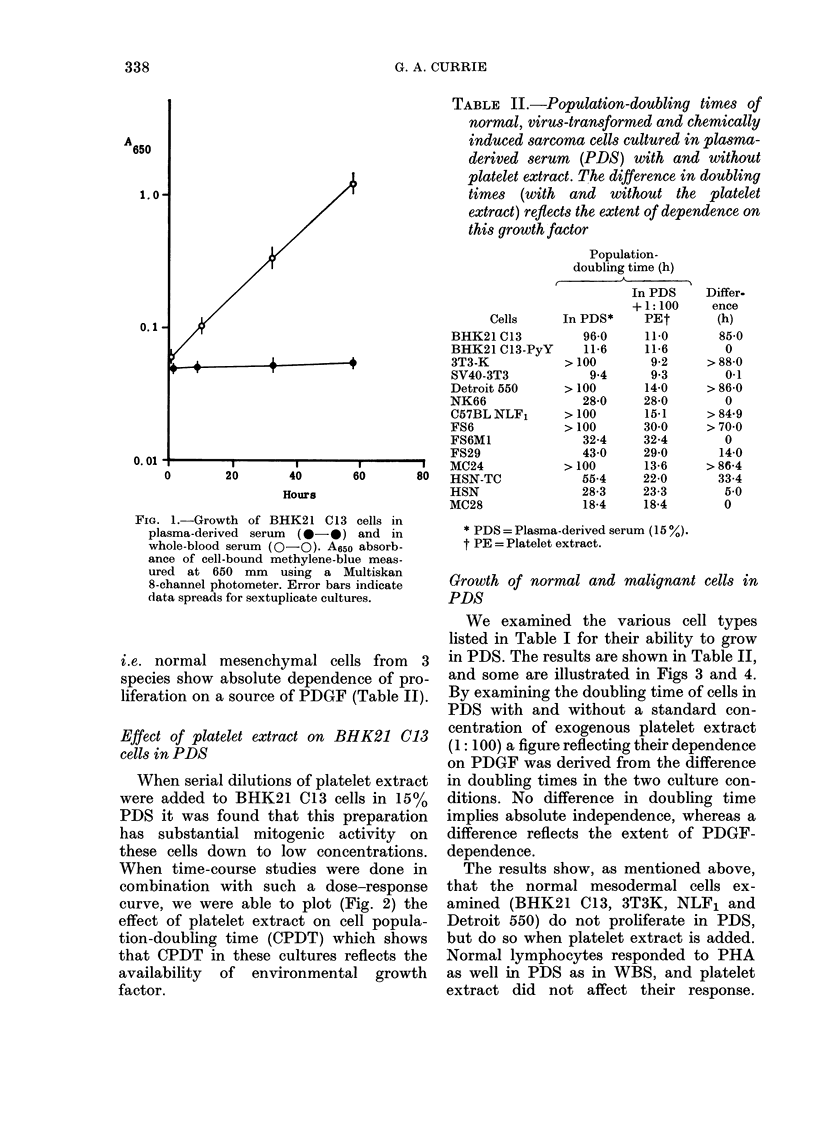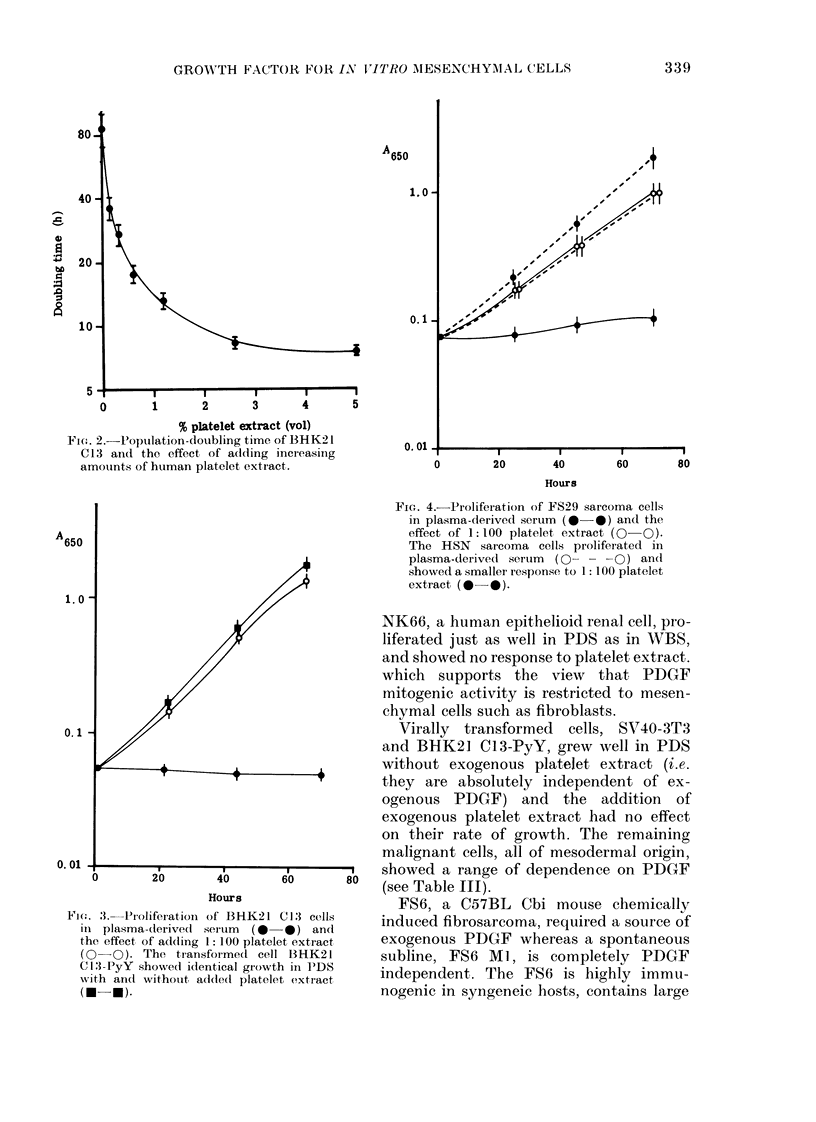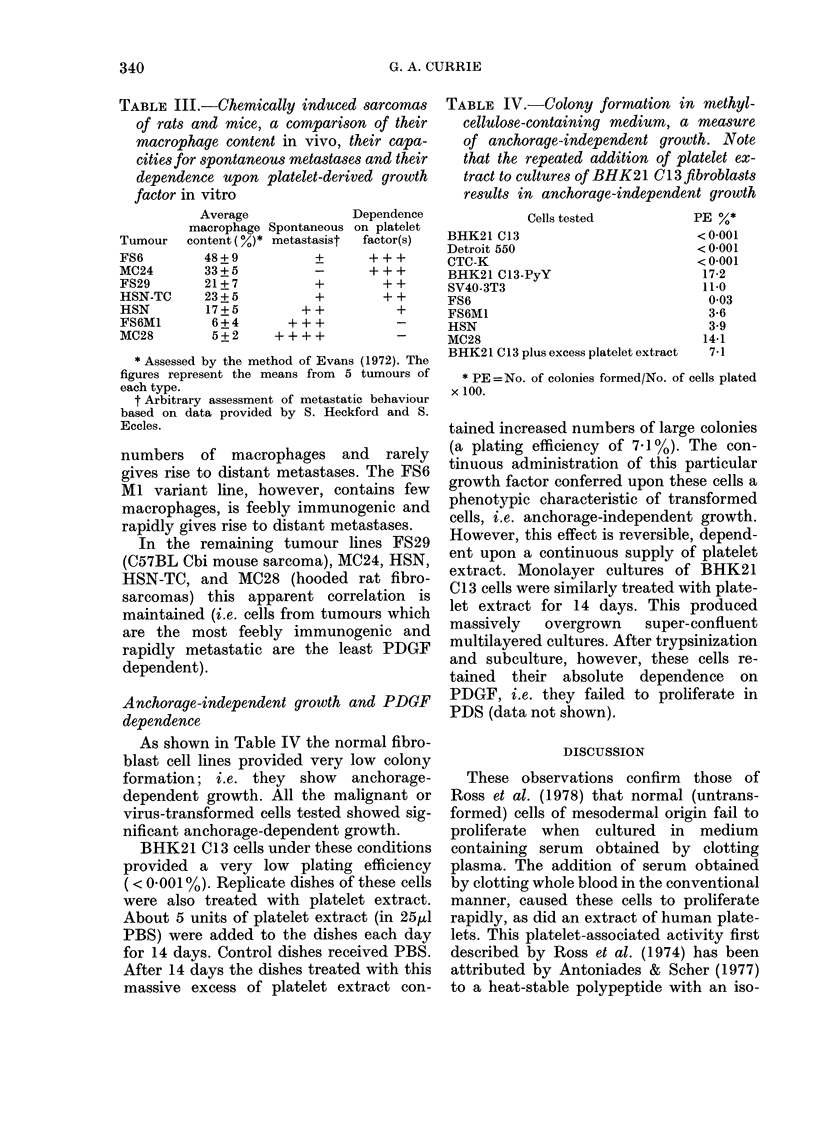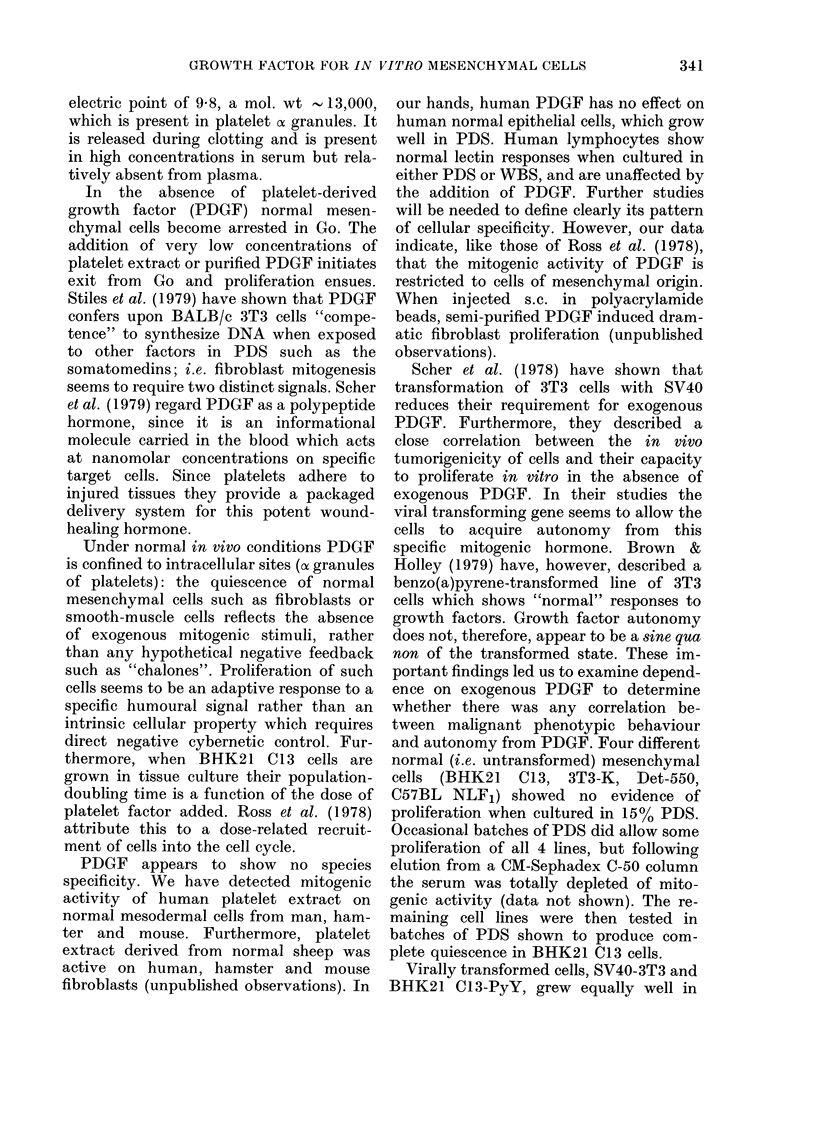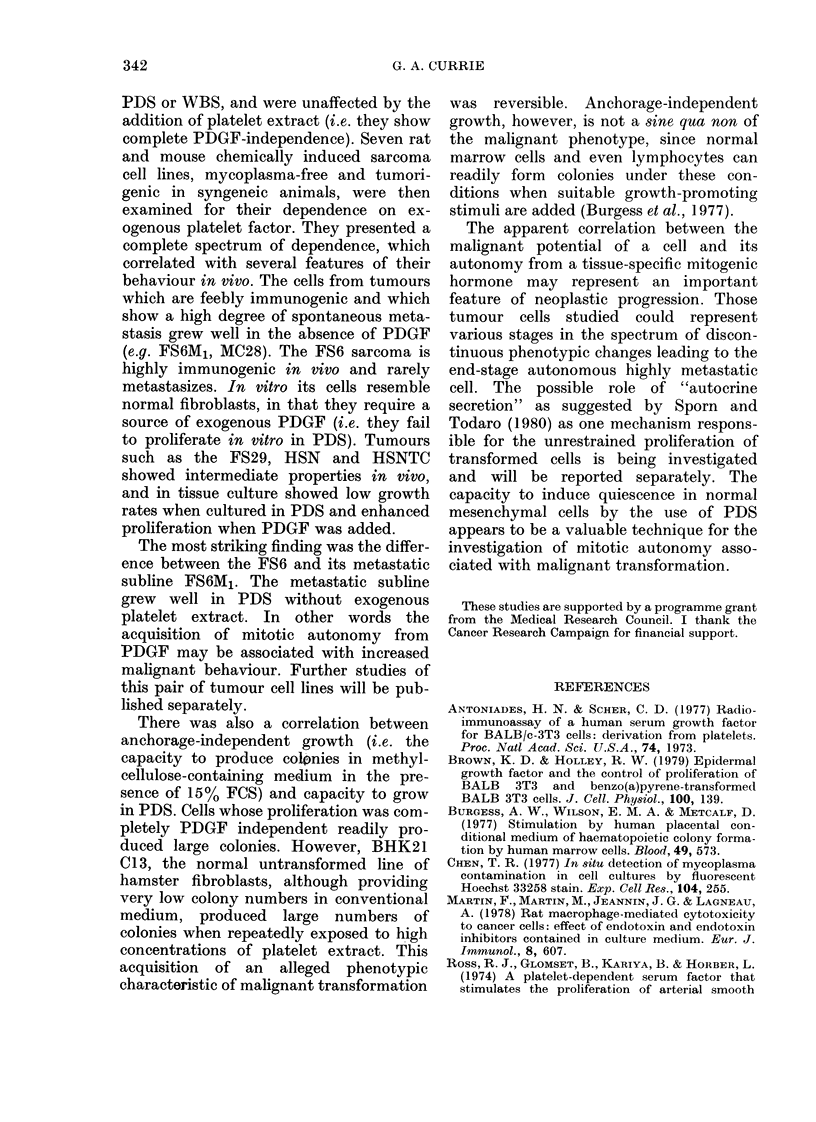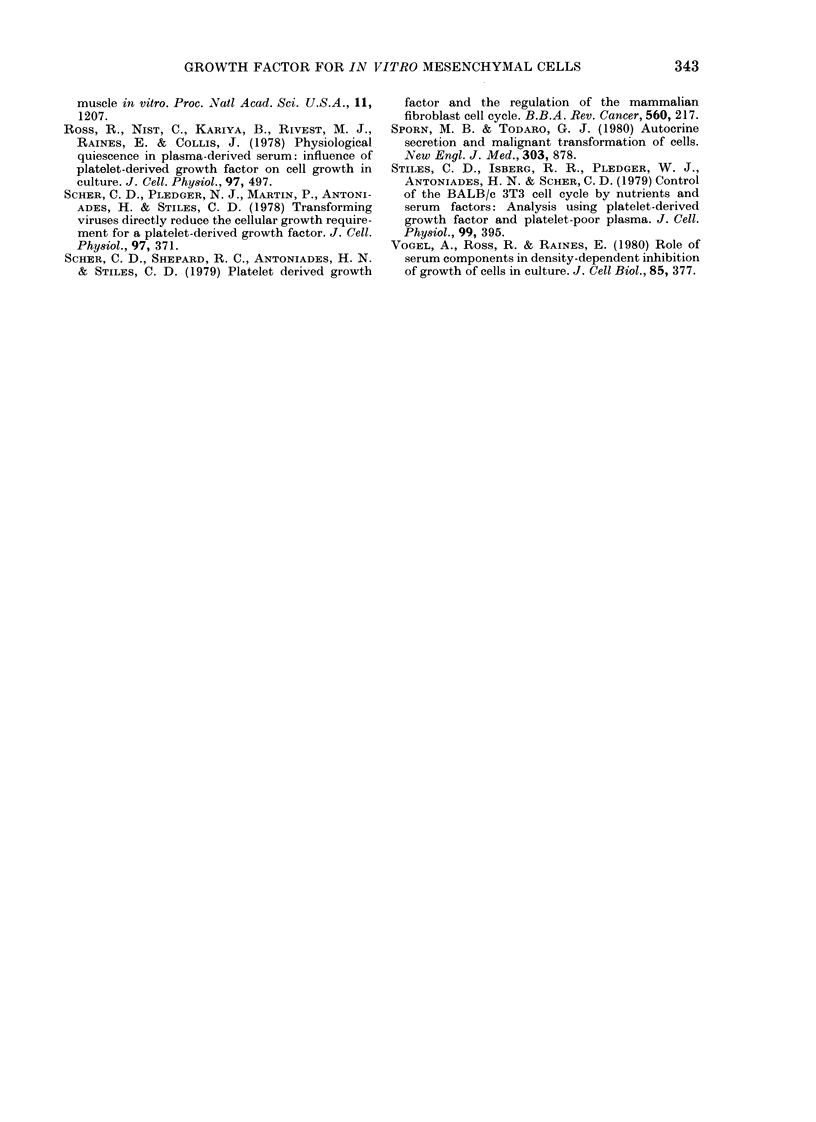# Platelet-derived growth-factor requirements for in vitro proliferation of normal and malignant mesenchymal cells.

**DOI:** 10.1038/bjc.1981.53

**Published:** 1981-03

**Authors:** G. A. Currie

## Abstract

Serum obtained by clotting whole blood contains a potent mitogen with apparent specificity for mesenchymal cells. This peptide wound-healing hormone, derived from platelets, is known as platelet-derived growth factor (PDGF). Serum obtained by clotting plasma contains no detectable growth-promoting activity for fibroblasts, and is therefore a valuable additive to culture medium for an examination of the autonomy of cells from exogenous PDGF. Fibroblasts from man, mouse and hamster remain mitotically quiescent in plasma-derived serum and proliferate only when a source of PDGF is added. Normal human kidney epithelial cells and human T-cells proliferate normally in plasma-derived serum, and are unaffected by the addition of PDGF. A range of virally transformed cells and malignant cells from chemically induced rodent sarcomas was tested for their proliferative capacity in plasma-derived serum and their response to exogenous PDGF. A complete spectrum of PDGF-dependence was revealed. Polyoma-transformed BHK21 cells and SV40-transformed 3T3 cells showed complete PDGF independence. Cells from 7 chemically induced rat or mouse sarcomas provided results which ranged from the FS6 (a C57BL Cbi mouse sarcoma which was completely PDGF dependent) to MC28 (a hooded rat sarcoma) which was completely PDGF independent. The dependence of proliferation of these cells on PDGF showed a close correlation with several features of their in vivo behaviour. Tumours which were non-immunogenic in syngeneic hosts, contained few host macrophages and produced a high incidence of spontaneous distant metastases provided PDGF-independent cells. Cells from highly immunogenic, macrophage-rich "non-metastasizing" tumours were on the other hand PDGF dependent and tumours of intermediate "malignancy" provided cells with partial autonomy from PDGF. An assay for anchorage-independent growth provided data which also correlated with autonomy from PDGF. However, daily addition of large amounts of PDGF to BHK21 C13 cells induced reversible anchorage independent growth. The value of plasma-derived serum for the investigation of the proliferative autonomy of malignant cells is emphasized.


*Br. J. Cancer* (1981) **43**, 335

### PLATELET-DERIVED GROWTH-FACTOR REQUIREMENTS FOR *IN VITRO* PROLIFERATION OF NORMAL AND MALIGNANT MESENCHYMAL CELLS

G. A. CURRIE

*From the Division of Tumour Immunology, Institute of Cancer Research, Sutton, Surrey*

Received 23 October 1980 Accepted 27 November 1980

**Summary.—Serum obtained by clotting whole blood contains a potent mitogen with apparent specificity for mesenchymal cells. This peptide wound-healing hormone, derived from platelets, is known as platelet-derived growth factor (PDGF). Serum obtained by clotting plasma contains no detectable growth-promoting activity for fibroblasts, and is therefore a valuable additive to culture medium for an examination of the autonomy of cells from exogenous PDGF. Fibroblasts from man, mouse and hamster remain mitotically quiescent in plasma-derived serum and proliferate only when a source of PDGF is added. Normal human kidney epithelial cells and human T-cells proliferate normally in plasma-derived serum, and are unaffected by the addition of PDGF.**

**A range of virally transformed cells and malignant cells from chemically induced rodent sarcomas was tested for their proliferative capacity in plasma-derived serum and their response to exogenous PDGF. A complete spectrum of PDGF-dependence was revealed. Polyoma-transformed BHK21 cells and SV40-transformed 3T3 cells showed complete PDGF independence. Cells from 7 chemically induced rat or mouse sarcomas provided results which ranged from the FS6 (a C57BL Cbi mouse sarcoma which was completely PDGF dependent) to MC28 (a hooded rat sarcoma) which was completely PDGF independent. The dependence of proliferation of these cells on PDGF showed a close correlation with several features of their *in vivo* behaviour. Tumours which were non-immunogenic in syngeneic hosts, contained few host macrophages and produced a high incidence of spontaneous distant metastases provided PDGF-independent cells. Cells from highly immunogenic, macrophage-rich "non-metastasizing" tumours were on the other hand PDGF dependent and tumours of intermediate "malignancy" provided cells with partial autonomy from PDGF.**

**An assay for anchorage-independent growth provided data which also correlated with autonomy from PDGF. However, daily addition of large amounts of PDGF to BHK21 C13 cells induced reversible anchorage independent growth. The value of plasma-derived serum for the investigation of the proliferative autonomy of malignant cells is emphasized.**

DURING CLOTTING, platelets release a potent mitogenic hormone with specificity for cells of mesodermal origin such as fibroblasts and smooth-muscle cells. This polypeptide, known as platelet-derived growth factor (PDGF) is thought to play an important role *in vivo* in wound healing (see review by Scher *et al.*, 1979). Normal fibroblasts will not proliferate in medium containing serum clotted in the absence of platelets, and remain quiescent for long periods. The subsequent addition of nanomolar amounts of purified PDGF initiates proliferation of such quiescent cultures. Density-dependent inhibition of growth of normal mesodermal cells can be attributed to exhaustion of PDGF from serum, as it can be reversed by the addition of PDGF (Vogel *et al.*, 1980).

Transformation of normal fibroblasts by oncogenic viruses such as SV40 reduces their requirements for exogeneous PDGF, and the loss of PDGF dependence appears to correlate with their *in vivo* tumorigenicity (Scher *et al.*, 1978). Furthermore, the capacity of these transformed cells to proliferate under conditions of serum restriction, and the relative absence of density-dependent growth inhibition in their cultures, reflects their decreased requirement for serum growth factors.

We describe here a series of experiments designed to examine the proliferative capacity of a series of normal, malignant (chemically induced) and virally transformed cells in culture medium depleted of PDGF, which reveals a relationship between the malignant phenotype of such cells and their proliferative dependence upon an exogenous source of PDGF.

### MATERIALS AND METHODS

### Cells

The cells studied are listed in Table I. They were all adapted to grow in RPM1 1640 containing 15% foetal bovine serum (FCS), 25mM HEPES and antibiotics. They were maintained at 37°C in 5% $CO_2$ in humid air as monolayer cultures, and were passaged frequently (using 0·1% trypsin) to maintain exponential growth. All cell lines were studied within 20 passages, and cultures were replenished from low passage stocks maintained in liquid $N_2$. They were routinely screened for mycoplasma contamination by fluorescence methods using Bisbenzimide H33258 (Chen, 1977) and were negative throughout these studies. The mouse and rat tumour lines were all shown at various times to be tumorigenic in syngeneic hosts.

### Sera

*Whole blood serum* (*WBS*).—20ml volumes of venous blood were taken from normal human volunteers and allowed to clot in plastic universal bottles containing glass beads. After clot retraction (37°C for 2 h) the serum was removed and heated to 56°C for 30 min and stored over liquid $N_2$.

*Plasma-derived serum* (*PDS*).—Similar volumes of blood were collected into chilled plastic universal bottles and these were then centrifuged at 4°C at 1000 g for 7 min. The plasma was then removed with a siliconized pipette and recentrifuged at 1000 *g* for 7 min. The plasma was then added to a universal bottle containing glass beads and allowed to clot at 37°C for 2 h. The fibrin clot was then removed by centrifugation and the PDS heated at 56°C for 30 min before storage over liquid $N_2$. WBS and PDS were thereby exposed to very similar conditions during preparation.

### Platelet extracts

Time-expired platelet preparations were obtained from the Haematology Department, Royal Marsden Hospital. The platelets were

TABLE I.—*List of cells used in these experiments*

| Cells | Description | Origin |
|---|---|---|
| BHK21 C13<br>BHK21 C13-PyY | "Normal" and polyoma virus-transformed baby hamster kidney | Flow Labs Ltd |
| 3T3-K and SV40-3T3 | "Normal" and SV40 virus-transformed mouse fibroblasts | Dr. L. Franks<br>ICRF Laboratories, London |
| Detroit 550 | Normal human fibroblast | Flow Labs Ltd |
| NK66 | Normal human kidney epithelial cell | Home grown |
| C57BL $NLF_1$ | Normal mouse lung fibroblast | Home grown |
| FS6 | Benzo(a)pyrene-induced mouse (C57BL Cbi) sarcoma | Home grown |
| $FS6M_1$ | Metastatic variant of FS6 | Home grown |
| FS29 | Benzo(a)pyrene-induced C57BL Cbi sarcoma | Home grown |
| MC24 | Methylcholanthrene-induced hooded rat sarcoma | Home grown |
| HSN-TC | Benzo(a)pyrene-induced hooded rat sarcoma subline | Home grown |
| HSN | Parent line of HSN-TC | Home grown |
| MC28 | Methylcholanthrene induced hooded rat sarcoma | Home grown |
| Normal human lymphocytes | Ficoll-Hypaque separated mononuclear cell suspensions | Normal healthy volunteers |

centrifuged at 1500 *g* for 20 min at 4°C and washed in 2 changes of acid citrate dextrose (ACD). The platelets from 5–10 u of blood were resuspended in 3 ml of phosphate-buffered saline (PBS) and rapidly frozen (using solid $CO_2$ and ethanol). They were then allowed to thaw and placed in a boiling water bath for 10 min. The precipitate was removed by centrifugation and the clear supernatant was dialysed overnight against PBS. It was then passed through a millipore 0·22$\mu$m filter and stored over liquid $N_2$. Since boiling removed most of the protein from these extracts the levels of protein were too low for conventional measurement. Batches of platelet extract were assayed in serial dilution on BHK21 C13 cells cultured in 15% PDS. One unit of growth factor was defined as the amount of platelet extract which produced 50% of maximal growth, assayed after 48 h exposure.

*Cell proliferation assay.*—This assay is a modification of the methylene-blue assay described by Martin *et al.* (1978). Cells were obtained from stock cultures by trypsinization and added in 100$\mu$l volumes to the wells of Microtest II plates (Falcon) using a Titertek 8-channel dispenser. To each well was added $1\text{–}5 \times 10^3$ cells (depending on the plating efficiency of the individual cell lines). The medium used was RPMI 1640 plus HEPES and antibiotics, containing 15% WBS or PDS. After 6–8h incubation at 37°C, when the cells had stuck and spread, one replicate plate was harvested and referred to as "time 0". Other plates were harvested at daily intervals thereafter.

For harvesting, the plates were slowly immersed in a tank of PBS at room temperature at an angle of $\sim$30° from the horizontal and then gently inverted and the wells drained by placing on a sheet of damp sponge (F. W. Woolworth).

This procedure was performed 3 times and then, to each well, 100 $\mu$l of 5% formol-saline were gently added. This gentle wash and fixation method produced no morphologically observable damage to cell monolayers. The cells were fixed for 30 min, when the formol-saline was removed by a flick of the wrist. To each well was then added 100 $\mu$l of 1% methylene blue in 0·01M borate at pH 8·5. The cells were stained for 30 min and the dye removed with another flick of the wrist. The plate was then thoroughly washed in 3 changes of borate buffer, inverted on to a stack of paper tissue and allowed to dry. When dry, 0·1 ml of 0·1N HCl was added to each well to elute the dye from the cells, and the plate was left for 10 min before gentle agitation by lateral shaking to mix the dye uniformly in each well.

The under surface of the plate was then carefully cleaned and read in a Multiskan (Titertek). This vertical path 8-channel spectrophotometer was used with an interference filter at 650 nm and provided printouts of optical density for each well of a 96-well plate. The Multiskan was blanked on the first row of wells, which in each plate contained the appropriate medium but no cells. The results were expressed as absorbance at 650 nm (A650). All experiments were performed in sextuplicate, and since this method provides extremely constant replicates, the error bars in the figures represent the total spread for 6 observations.

*Anchorage-independent growth.*—Cells obtained from stock cultures were seeded in complete medium containing 1·2% methylcellulose (Methocel A4M) into triplicate 60mm plastic dishes at $10^3$ and $10^4$ cells per dish. The dishes contained a thin coating (2 ml per dish) of 0·9% low-gelling-temperature agarose in complete medium. After plating, any dish with clumped cells was discarded.

After incubation for 14 days the number of observable colonies was counted under a low-power stereo microscope; only "large" colonies were counted, *i.e.* colonies judged to contain $>50$ cells. The results are presented as "plating efficiencies", *i.e.* the number of observable colonies as a percentage of the number of cells added per dish.

### RESULTS

### *Growth of normal cells in PDS* vs *WBS*

BHK21 C13 were used in many of these studies. These untransformed baby hamster kidney cells proliferated rapidly in 15% WBS. However, when cultured in 15% PDS they failed to divide (see Fig. 1). The addition of platelet extract to such cultures in PDS, however, readily stimulated proliferation.

Similar results were obtained with 3T3K mouse cells, Detroit 550 human fibroblasts and $NLF_1$ mouse fibroblasts;

FIG. 1.—Growth of BHK21 C13 cells in plasma-derived serum (●—●) and in whole-blood serum (○—○). $A_{650}$ absorbance of cell-bound methylene-blue measured at 650 mm using a Multiskan 8-channel photometer. Error bars indicate data spreads for sextuplicate cultures.

*i.e.* normal mesenchymal cells from 3 species show absolute dependence of proliferation on a source of PDGF (Table II).

*Effect of platelet extract on BHK21 C13 cells in PDS*

When serial dilutions of platelet extract were added to BHK21 C13 cells in 15% PDS it was found that this preparation has substantial mitogenic activity on these cells down to low concentrations. When time-course studies were done in combination with such a dose–response curve, we were able to plot (Fig. 2) the effect of platelet extract on cell population-doubling time (CPDT) which shows that CPDT in these cultures reflects the availability of environmental growth factor.

TABLE II.—*Population-doubling times of normal, virus-transformed and chemically induced sarcoma cells cultured in plasma-derived serum (PDS) with and without platelet extract. The difference in doubling times (with and without the platelet extract) reflects the extent of dependence on this growth factor*

| | Population-doubling time (h) | | |
|---|---|---|---|
| Cells | In PDS* | In PDS +1:100 PE† | Difference (h) |
| BHK21 C13 | 96·0 | 11·0 | 85·0 |
| BHK21 C13-PyY | 11·6 | 11·6 | 0 |
| 3T3-K | >100 | 9·2 | >88·0 |
| SV40-3T3 | 9·4 | 9·3 | 0·1 |
| Detroit 550 | >100 | 14·0 | >86·0 |
| NK66 | 28·0 | 28·0 | 0 |
| C57BL NLF$_1$ | >100 | 15·1 | >84·9 |
| FS6 | >100 | 30·0 | >70·0 |
| FS6M1 | 32·4 | 32·4 | 0 |
| FS29 | 43·0 | 29·0 | 14·0 |
| MC24 | >100 | 13·6 | >86·4 |
| HSN-TC | 55·4 | 22·0 | 33·4 |
| HSN | 28·3 | 23·3 | 5·0 |
| MC28 | 18·4 | 18·4 | 0 |

* PDS = Plasma-derived serum (15%).

† PE = Platelet extract.

*Growth of normal and malignant cells in PDS*

We examined the various cell types listed in Table I for their ability to grow in PDS. The results are shown in Table II, and some are illustrated in Figs 3 and 4. By examining the doubling time of cells in PDS with and without a standard concentration of exogenous platelet extract (1:100) a figure reflecting their dependence on PDGF was derived from the difference in doubling times in the two culture conditions. No difference in doubling time implies absolute independence, whereas a difference reflects the extent of PDGF-dependence.

The results show, as mentioned above, that the normal mesodermal cells examined (BHK21 C13, 3T3K, NLF$_1$ and Detroit 550) do not proliferate in PDS, but do so when platelet extract is added. Normal lymphocytes responded to PHA as well in PDS as in WBS, and platelet extract did not affect their response.

FIG. 2.—Population-doubling time of BHK21 C13 and the effect of adding increasing amounts of human platelet extract.

FIG. 3.—Proliferation of BHK21 C13 cells in plasma-derived serum (●—●) and the effect of adding 1: 100 platelet extract (○—○). The transformed cell BHK21 C13-PyY showed identical growth in PDS with and without added platelet extract (■—■).

FIG. 4.—Proliferation of FS29 sarcoma cells in plasma-derived serum (●—●) and the effect of 1: 100 platelet extract (○—○). The HSN sarcoma cells proliferated in plasma-derived serum (○– – –○) and showed a smaller response to 1: 100 platelet extract (●—●).

NK66, a human epithelioid renal cell, proliferated just as well in PDS as in WBS, and showed no response to platelet extract. which supports the view that PDGF mitogenic activity is restricted to mesenchymal cells such as fibroblasts.

Virally transformed cells, SV40-3T3 and BHK21 C13-PyY, grew well in PDS without exogenous platelet extract (*i.e.* they are absolutely independent of exogenous PDGF) and the addition of exogenous platelet extract had no effect on their rate of growth. The remaining malignant cells, all of mesodermal origin, showed a range of dependence on PDGF (see Table III).

FS6, a C57BL Cbi mouse chemically induced fibrosarcoma, required a source of exogenous PDGF whereas a spontaneous subline, FS6 M1, is completely PDGF independent. The FS6 is highly immunogenic in syngeneic hosts, contains large

TABLE III.—*Chemically induced sarcomas of rats and mice, a comparison of their macrophage content* in vivo, *their capacities for spontaneous metastases and their dependence upon platelet-derived growth factor* in vitro

| Tumour | Average macrophage content (%)* | Spontaneous metastasis† | Dependence on platelet factor(s) |
|---|---|---|---|
| FS6 | 48±9 | ± | +++ |
| MC24 | 33±5 | − | +++ |
| FS29 | 21±7 | + | ++ |
| HSN-TC | 23±5 | + | ++ |
| HSN | 17±5 | ++ | + |
| FS6M1 | 6±4 | +++ | − |
| MC28 | 5±2 | ++++ | − |

* Assessed by the method of Evans (1972). The figures represent the means from 5 tumours of each type.

† Arbitrary assessment of metastatic behaviour based on data provided by S. Heckford and S. Eccles.

numbers of macrophages and rarely gives rise to distant metastases. The FS6 M1 variant line, however, contains few macrophages, is feebly immunogenic and rapidly gives rise to distant metastases.

In the remaining tumour lines FS29 (C57BL Cbi mouse sarcoma), MC24, HSN, HSN-TC, and MC28 (hooded rat fibrosarcomas) this apparent correlation is maintained (*i.e.* cells from tumours which are the most feebly immunogenic and rapidly metastatic are the least PDGF dependent).

*Anchorage-independent growth and PDGF dependence*

As shown in Table IV the normal fibroblast cell lines provided very low colony formation; *i.e.* they show anchorage-dependent growth. All the malignant or virus-transformed cells tested showed significant anchorage-dependent growth.

BHK21 C13 cells under these conditions provided a very low plating efficiency (<0·001%). Replicate dishes of these cells were also treated with platelet extract. About 5 units of platelet extract (in 25μl PBS) were added to the dishes each day for 14 days. Control dishes received PBS. After 14 days the dishes treated with this massive excess of platelet extract con-

TABLE IV.—*Colony formation in methylcellulose-containing medium, a measure of anchorage-independent growth. Note that the repeated addition of platelet extract to cultures of BHK*21 *C*13 *fibroblasts results in anchorage-independent growth*

| Cells tested | PE %* |
|---|---|
| BHK21 C13 | <0·001 |
| Detroit 550 | <0·001 |
| CTC-K | <0·001 |
| BHK21 C13-PyY | 17·2 |
| SV40-3T3 | 11·0 |
| FS6 | 0·03 |
| FS6M1 | 3·6 |
| HSN | 3·9 |
| MC28 | 14·1 |
| BHK21 C13 plus excess platelet extract | 7·1 |

* PE = No. of colonies formed/No. of cells plated ×100.

tained increased numbers of large colonies (a plating efficiency of 7·1%). The continuous administration of this particular growth factor conferred upon these cells a phenotypic characteristic of transformed cells, *i.e.* anchorage-independent growth. However, this effect is reversible, dependent upon a continuous supply of platelet extract. Monolayer cultures of BHK21 C13 cells were similarly treated with platelet extract for 14 days. This produced massively overgrown super-confluent multilayered cultures. After trypsinization and subculture, however, these cells retained their absolute dependence on PDGF, *i.e.* they failed to proliferate in PDS (data not shown).

### DISCUSSION

These observations confirm those of Ross *et al.* (1978) that normal (untransformed) cells of mesodermal origin fail to proliferate when cultured in medium containing serum obtained by clotting plasma. The addition of serum obtained by clotting whole blood in the conventional manner, caused these cells to proliferate rapidly, as did an extract of human platelets. This platelet-associated activity first described by Ross *et al.* (1974) has been attributed by Antoniades & Scher (1977) to a heat-stable polypeptide with an isoelectric point of 9·8, a mol. wt ~13,000, which is present in platelet α granules. It is released during clotting and is present in high concentrations in serum but relatively absent from plasma.

In the absence of platelet-derived growth factor (PDGF) normal mesenchymal cells become arrested in Go. The addition of very low concentrations of platelet extract or purified PDGF initiates exit from Go and proliferation ensues. Stiles *et al.* (1979) have shown that PDGF confers upon BALB/c 3T3 cells "competence" to synthesize DNA when exposed to other factors in PDS such as the somatomedins; *i.e.* fibroblast mitogenesis seems to require two distinct signals. Scher *et al.* (1979) regard PDGF as a polypeptide hormone, since it is an informational molecule carried in the blood which acts at nanomolar concentrations on specific target cells. Since platelets adhere to injured tissues they provide a packaged delivery system for this potent wound-healing hormone.

Under normal *in vivo* conditions PDGF is confined to intracellular sites (α granules of platelets): the quiescence of normal mesenchymal cells such as fibroblasts or smooth-muscle cells reflects the absence of exogenous mitogenic stimuli, rather than any hypothetical negative feedback such as "chalones". Proliferation of such cells seems to be an adaptive response to a specific humoural signal rather than an intrinsic cellular property which requires direct negative cybernetic control. Furthermore, when BHK21 C13 cells are grown in tissue culture their population-doubling time is a function of the dose of platelet factor added. Ross *et al.* (1978) attribute this to a dose-related recruitment of cells into the cell cycle.

PDGF appears to show no species specificity. We have detected mitogenic activity of human platelet extract on normal mesodermal cells from man, hamter and mouse. Furthermore, platelet extract derived from normal sheep was active on human, hamster and mouse fibroblasts (unpublished observations). In our hands, human PDGF has no effect on human normal epithelial cells, which grow well in PDS. Human lymphocytes show normal lectin responses when cultured in either PDS or WBS, and are unaffected by the addition of PDGF. Further studies will be needed to define clearly its pattern of cellular specificity. However, our data indicate, like those of Ross *et al.* (1978), that the mitogenic activity of PDGF is restricted to cells of mesenchymal origin. When injected s.c. in polyacrylamide beads, semi-purified PDGF induced dramatic fibroblast proliferation (unpublished observations).

Scher *et al.* (1978) have shown that transformation of 3T3 cells with SV40 reduces their requirement for exogenous PDGF. Furthermore, they described a close correlation between the *in vivo* tumorigenicity of cells and their capacity to proliferate *in vitro* in the absence of exogenous PDGF. In their studies the viral transforming gene seems to allow the cells to acquire autonomy from this specific mitogenic hormone. Brown & Holley (1979) have, however, described a benzo(a)pyrene-transformed line of 3T3 cells which shows "normal" responses to growth factors. Growth factor autonomy does not, therefore, appear to be a *sine qua non* of the transformed state. These important findings led us to examine dependence on exogenous PDGF to determine whether there was any correlation between malignant phenotypic behaviour and autonomy from PDGF. Four different normal (*i.e.* untransformed) mesenchymal cells (BHK21 C13, 3T3-K, Det-550, C57BL $NLF_1$) showed no evidence of proliferation when cultured in 15% PDS. Occasional batches of PDS did allow some proliferation of all 4 lines, but following elution from a CM-Sephadex C-50 column the serum was totally depleted of mitogenic activity (data not shown). The remaining cell lines were then tested in batches of PDS shown to produce complete quiescence in BHK21 C13 cells.

Virally transformed cells, SV40-3T3 and BHK21 C13-PyY, grew equally well in

PDS or WBS, and were unaffected by the addition of platelet extract (*i.e.* they show complete PDGF-independence). Seven rat and mouse chemically induced sarcoma cell lines, mycoplasma-free and tumorigenic in syngeneic animals, were then examined for their dependence on exogenous platelet factor. They presented a complete spectrum of dependence, which correlated with several features of their behaviour *in vivo*. The cells from tumours which are feebly immunogenic and which show a high degree of spontaneous metastasis grew well in the absence of PDGF (*e.g.* $FS6M_1$, MC28). The FS6 sarcoma is highly immunogenic *in vivo* and rarely metastasizes. *In vitro* its cells resemble normal fibroblasts, in that they require a source of exogenous PDGF (*i.e.* they fail to proliferate *in vitro* in PDS). Tumours such as the FS29, HSN and HSNTC showed intermediate properties *in vivo*, and in tissue culture showed low growth rates when cultured in PDS and enhanced proliferation when PDGF was added.

The most striking finding was the difference between the FS6 and its metastatic subline $FS6M_1$. The metastatic subline grew well in PDS without exogenous platelet extract. In other words the acquisition of mitotic autonomy from PDGF may be associated with increased malignant behaviour. Further studies of this pair of tumour cell lines will be published separately.

There was also a correlation between anchorage-independent growth (*i.e.* the capacity to produce colonies in methylcellulose-containing medium in the presence of 15% FCS) and capacity to grow in PDS. Cells whose proliferation was completely PDGF independent readily produced large colonies. However, BHK21 C13, the normal untransformed line of hamster fibroblasts, although providing very low colony numbers in conventional medium, produced large numbers of colonies when repeatedly exposed to high concentrations of platelet extract. This acquisition of an alleged phenotypic characteristic of malignant transformation was reversible. Anchorage-independent growth, however, is not a *sine qua non* of the malignant phenotype, since normal marrow cells and even lymphocytes can readily form colonies under these conditions when suitable growth-promoting stimuli are added (Burgess *et al.*, 1977).

The apparent correlation between the malignant potential of a cell and its autonomy from a tissue-specific mitogenic hormone may represent an important feature of neoplastic progression. Those tumour cells studied could represent various stages in the spectrum of discontinuous phenotypic changes leading to the end-stage autonomous highly metastatic cell. The possible role of "autocrine secretion" as suggested by Sporn and Todaro (1980) as one mechanism responsible for the unrestrained proliferation of transformed cells is being investigated and will be reported separately. The capacity to induce quiescence in normal mesenchymal cells by the use of PDS appears to be a valuable technique for the investigation of mitotic autonomy associated with malignant transformation.

These studies are supported by a programme grant from the Medical Research Council. I thank the Cancer Research Campaign for financial support.

### REFERENCES

ANTONIADES, H. N. & SCHER, C. D. (1977) Radioimmunoassay of a human serum growth factor for BALB/c-3T3 cells: derivation from platelets. *Proc. Natl Acad. Sci. U.S.A.*, **74**, 1973.

BROWN, K. D. & HOLLEY, R. W. (1979) Epidermal growth factor and the control of proliferation of BALB 3T3 and benzo(a)pyrene-transformed BALB 3T3 cells. *J. Cell. Physiol.*, **100**, 139.

BURGESS, A. W., WILSON, E. M. A. & METCALF, D. (1977) Stimulation by human placental conditional medium of haematopoietic colony formation by human marrow cells. *Blood*, **49**, 573.

CHEN, T. R. (1977) *In situ* detection of mycoplasma contamination in cell cultures by fluorescent Hoechst 33258 stain. *Exp. Cell Res.*, **104**, 255.

MARTIN, F., MARTIN, M., JEANNIN, J. G. & LAGNEAU, A. (1978) Rat macrophage-mediated cytotoxicity to cancer cells: effect of endotoxin and endotoxin inhibitors contained in culture medium. *Eur. J. Immunol.*, **8**, 607.

ROSS, R. J., GLOMSET, B., KARIYA, B. & HORBER, L. (1974) A platelet-dependent serum factor that stimulates the proliferation of arterial smooth muscle *in vitro*. *Proc. Natl Acad. Sci. U.S.A.*, **11**, 1207.

ROSS, R., NIST, C., KARIYA, B., RIVEST, M. J., RAINES, E. & COLLIS, J. (1978) Physiological quiescence in plasma-derived serum: influence of platelet-derived growth factor on cell growth in culture. *J. Cell. Physiol.*, **97**, 497.

SCHER, C. D., PLEDGER, N. J., MARTIN, P., ANTONIADES, H. & STILES, C. D. (1978) Transforming viruses directly reduce the cellular growth requirement for a platelet-derived growth factor. *J. Cell. Physiol.*, **97**, 371.

SCHER, C. D., SHEPARD, R. C., ANTONIADES, H. N. & STILES, C. D. (1979) Platelet derived growth factor and the regulation of the mammalian fibroblast cell cycle. *B.B.A. Rev. Cancer*, **560**, 217.

SPORN, M. B. & TODARO, G. J. (1980) Autocrine secretion and malignant transformation of cells. *New Engl. J. Med.*, **303**, 878.

STILES, C. D., ISBERG, R. R., PLEDGER, W. J., ANTONIADES, H. N. & SCHER, C. D. (1979) Control of the BALB/c 3T3 cell cycle by nutrients and serum factors: Analysis using platelet-derived growth factor and platelet-poor plasma. *J. Cell. Physiol.*, **99**, 395.

VOGEL, A., ROSS, R. & RAINES, E. (1980) Role of serum components in density-dependent inhibition of growth of cells in culture. *J. Cell Biol.*, **85**, 377.